# Orthodontically Induced External Apical Root Resorption in Class II Malocclusion

**DOI:** 10.1155/2021/8290429

**Published:** 2021-12-22

**Authors:** Praveen Kumar Reddy Karnati, Priyank Seth, Ahmad Termizi Bin Zamzuri, Payal Tharwani

**Affiliations:** ^1^Faculty of Dentistry, SEGi University, Kota Damansara, Selangor, Malaysia; ^2^Faculty of Dentistry, Bhitai Dental College, Mirpurkhas, Pakistan

## Abstract

Orthodontic-induced external apical root resorption is one of the idiopathic phenomena as an effect, with force generated through mechanotherapy as the cause and the biological tissues with their diversified variations as witness. It is also classified as iatrogenic as a result of indeterminate application of orthodontic forces with subconscious appreciation of the existing underlying conditions. Numerous factors were identified to relate to this irreversible pathologic condition, but none were proven scientifically. Genetics and salivary markers have proved the reliability with time, but the application became insignificant limiting mostly to the research field. Different assessment methods were also identified to clinically diagnose it both subjectively and objectively. Mostly, it is identified through routine radiographic stage records like orthopantomogram or certain prediction radiographs for root resorption probability assessment like in this case. This case report discusses one such encounter which was experienced after stage 1 and 2 mechanics involving quite a few teeth. Considering the biotype of the individual and tooth morphology, the ongoing treatment was terminated and recovery measures were briefed to uplift the self-esteem of the individual. Furthermore, the prognosis is compromised to be very poor with unpredictability to any other treatment modalities.

## 1. Introduction

Orthodontic-induced external apical root resorption (OIEARR) is one of the complications of orthodontic treatment besides other disadvantages [1], due to idiopathic causes and conditions. OIEARR is identified as a permanent shortening of the tooth root structure involving one or more teeth, generally the maxillary central and lateral incisors being mostly affected [2]. The degree of severity can be manifested proportionally with the type of force and magnitude generated by orthodontic appliances and classified as pathological in nature [3, 4]. More than a third of individuals treated with fixed appliances have shown greater than 3 mm of root length resorption, whereas in 2% to 5% cases up to 5 mm of root resorption (RR) has been witnessed thereby compromising the function and survival of the tooth involved [5, 6].

Due to its numerous ways of etiopathogenesis, the clinical manifestations also remain highly variable, suggesting new insights for additional diagnostic tools and markers. Having just a small sample of saliva and checking it for potential diagnostic protein biomarkers for orthodontic-induced inflammatory root resorption could prevent such unfavorable situations [7]. Routine pretreatment radiographic assessment and random intraoral periapical radiographs (IOPAR) have proved efficient, as they were prospective in ruling out the contribution of different root anomalies as well as identification of OIEARR during treatment with minimal compromise [8–10].

The present case report discusses one such anticipated occurrence of OIEARR in a class II malocclusion out of exemplary precautions which compromised the fulfillment of objectives leading to discontinuation of treatment.

## 2. Case Presentation

### 2.1. Clinical Examination

**Figure 1:**
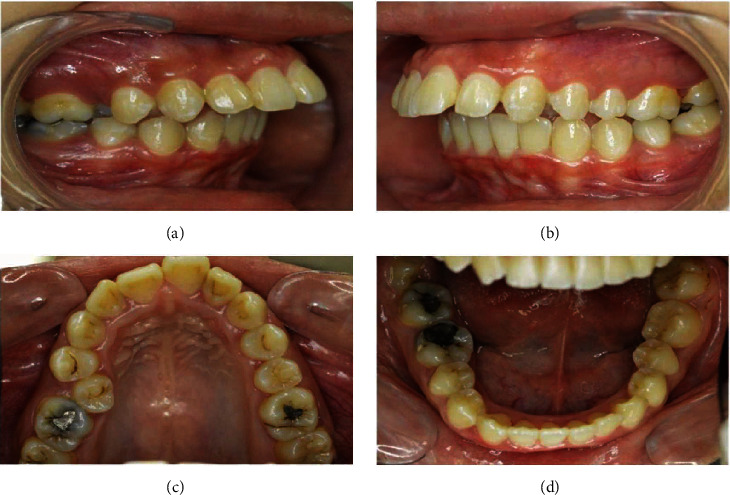
(a–d) Pretreatment intraoral photographs.

A 19-year-old female patient presented with inability to bite with her front teeth and some speech difficulty. She had an unremarkable medical history. Clinically, she was a thin biotype with symmetrical extraoral face, convex profile, and increased vertical proportions. Her lips were potentially incompetent with acute nasolabial angle, shallow mentolabial sulcus, and simple tongue thrust habit. This was further complicated by increased overjet of 11 mm, hyperdivergent jaw bases with anterior open bite by 1 mm, narrow arches, palatally erupted #14, upper dental midline shift towards the right, cuspal class II relation on the right and full tooth class II on the left side, class II division 1 incisor relation, and circum-oral muscular hyperactivity on swallow (Figures 1(a)–1(d)). The temporomandibular joint function was asymptomatic with evident jaw deviation on maximum opening and spontaneous return to normal on closure.

### 2.2. Radiographic Examination

**Figure 2:**
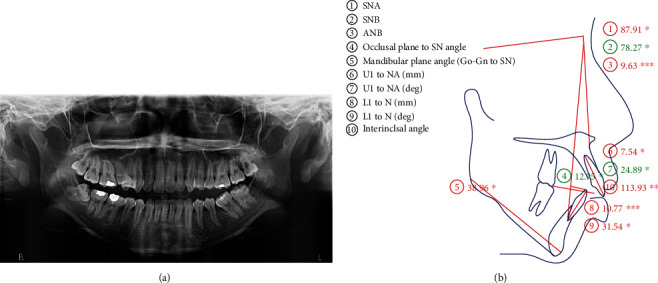
(a) Pretreatment OPG and (b) lateral cephalogram tracing with values.

Orthopantomogram (OPG) (Gendex Orthoralix 9200 DDE, Gendex Dental Systems, 901 W Oakton St., Des Plaines IL 60018-184) revealed certain teeth with pipette-shaped, pointed apical third root contours with few others dilacerated and rectangular morphology. Generalized horizontal bone loss was also identified in the upper and lower posterior regions (Figure 2(a)). The skeletal relation was class II contributed by severe prognathic maxilla and mild retrognathic mandible. The maxillary dentition had a neutral compensation with protrusion, while the mandibular teeth had unfavorable compensation with both inclination and protrusion which were measured using online WebCeph software application (Figure 2(b)).

### 2.3. Treatment Plan

Establishing her treatment prognosis at average outcome, camouflage treatment was planned with the patient's consent and disobedience of the surgical approach, in achieving optimal structural balance and functional efficiency with esthetic harmony. Damon MBT self-ligating 0.022 × 0.028^″^ bracket prescription was bonded from the second molar to the second molar in both arches. Damon-Q (ORMCO, California, USA) was selected for her by estimating its passive self-ligation property with the initial 2 stages generating less friction thereby reducing the resistance between the root surface and bone to prevent root resorption. Symmetric extractions of #14 and #24 were done to restore the overjet with some compromise by 2-3 mm along with nonextraction in the opposing arch. Subsequently, a little more space generation was planned by widening of the arches as well as molar distalization of the upper left molars to establish a cuspal class II molar relation and simultaneously correcting the midline and for bilateral intrusion of the posterior teeth to restore the optimal vertical proportion. For this, #18 and #28 were also included in therapeutic extractions alongside the premolars. Infrazygomatic cortical miniscrew assistance was considered to reinforce the anchorage.

**Figure 3:**
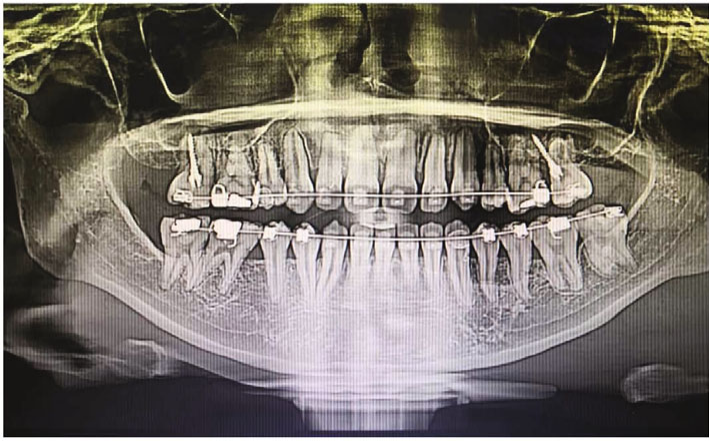
Midtreatment OPG showing OIEARR varying from moderate to severe in maxillary teeth and mild in mandibular teeth.

**Figure 4:**
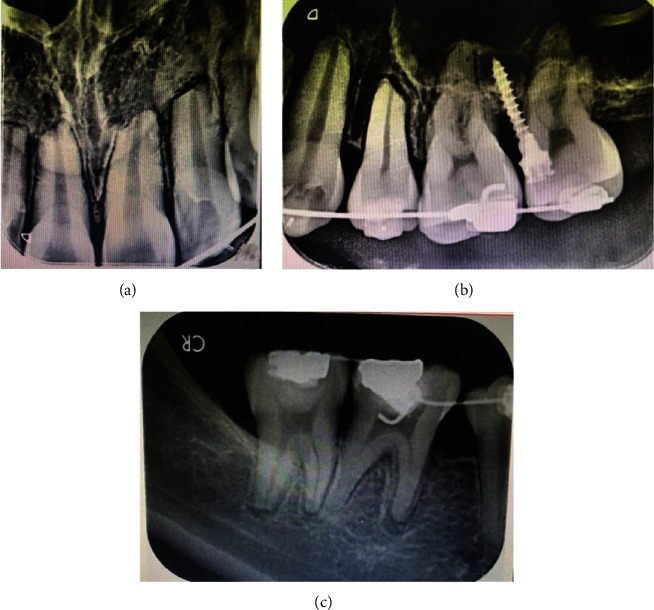
Intraoral periapical radiographs of (a) maxillary incisors, (b) maxillary left posterior teeth, and (c) mandibular right posterior teeth showing severe OIEARR.

**Figure 5:**
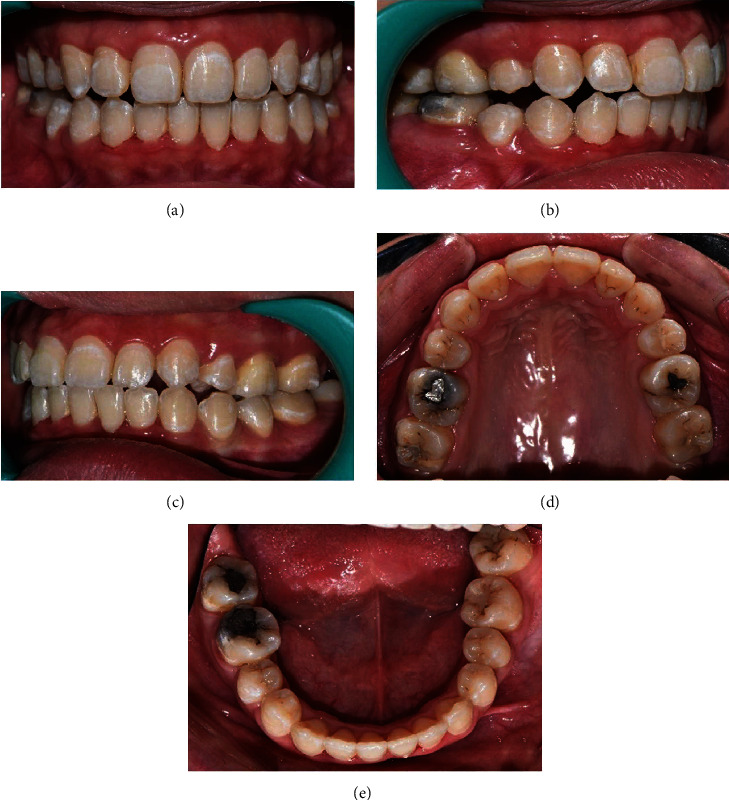
(a–e) Midtreatment intraoral photographs.

Stage I treatment of aligning and leveling was carried for 15 months with critical type A anchorage over 0.014^″^, 0.016, and 0.014 × 0.025^″^ thermal Nickel Titanium (T-NiTi) archwires followed by 0.017 × 0.025^″^ and 0.019 × 0.025^″^ T-NiTi ending with 0.019 × 0.025^″^ stainless steel (SS) archwires (ORMCO, California, USA). The duration between the appointments was also delayed due to multiple time brackets and buccal tube dislodgement along with archwire changes to maintain optimal conditions to avoid any root resorption (Table 1). Stage II objective of space closure was carried out for 11 months on an optimal reverse curve of Spee with active tie back, reinforced with intermaxillary class II elastics of 2-ounce force to control the vertical relation of incisors. Overjet was effectively addressed, and a stage II OPG was taken to proceed further with molar distalization, intrusion of posterior teeth, and midline correction with infrazygomatic cortical mini-implants assistance. The OPG and (IOPAR) revealed multiple teeth root resorption both in the maxillary anterior and posterior teeth as well as in certain mandibular teeth (Figure 3) after 26 months of initiation, compromising further treatment and discontinuation of all other planned procedures. The most affected were maxillary incisors and premolars around 4 mm followed by canines, molars (Figure 4), and the least being mandibular teeth ranging from 0 to 3 mm, as no major mechanics was involved. Seventy percent of the objectives were achieved by the time of radiographic root resorption appreciated. Clinically, the intraoral picture was healthy and absolutely without any signs of root resorption (Figure 5) which was occurring within the periodontium. The appliance was debonded on patient's request, and education was given for further maintenance and support over managing of the incidence. Upper and lower essix retainers were issued with three 3-month interval review check-up appointments to monitor the relapse incidence of orthodontic treatment results as well as progression of root resorption. Long-term retention was advised.

## 3. Discussion

Orthodontic-induced external apical root resorption (OIEARR) is a globally prevalent undesirable pathological condition without any discrimination even today that is widely documented by researchers [3]. It is genetic predisposition, ever since published by Newman et al. (1975) with family clustering of inexplicable inheritance patterns among its diversity, which was later supported with one of such findings by Al-Qawasmi et al. [11] related to proinflammatory cytokines like IL-1A and IL-1B on IL-1 gene cluster on human chromosome 2q13 substantiating the clinical perception that there is more to root resorption than amount of force or type of appliance used. The radiographic findings of thin, pointed, barrel-shaped, and dilacerated root morphology as an individual predisposing factor and recognizable radiographic marker in the prediction of OIEARR in this case were also coincidental in Brezniak and Wasserstein [9] and Geraldo de O et al. [12] study who also found a greater correlation of root resorption with similar findings after the orthodontic treatment.

Among the types of malocclusions, class II is mostly reported for orthodontic intervention alongside the complexity. Similarly, increased overjet is also credited to the incidence of root resorption. Brin et al. [13] in their retrospective study found interesting facts on increased overjet with one versus two-phase treatment relatively attributing it to the total treatment duration in achieving the objectives. They disclosed significant results with the magnitude of overjet reduction and the severity of root resorption being proportionally increasing from moderate to severe OIEARR, likewise varying between one versus 2 phase treatment accordingly. As the growth spurt was completed in this case, camouflage treatment was elected by dental compensation of skeletal malrelation. Janson et al. [14] found the incidence of OIEARR in both nonextraction and extraction treatment approaches ranging from mild to severe with clinically insignificant difference in results among the practices which was correlated with this condition. Visible changes can be witnessed as early as within 6 months during the aligning and leveling phase or by the end of the space closure phase through stage radiographs.

Damon self-ligating bracket prescription “Damon-Q2” was selected to reduce the incidence of root resorption as it is passive and its low friction property would be advantageous for stage I and stage II mechanics thereby benefitting the patient in minimizing the occurrence of OIEARR compared to conventional bracket prescription. On the contrary, studies have proven no such significant differences existed with root resorption between the prescriptions. In their study on class I malocclusion, mild to moderate crowding, and nonextraction, Handem et al. reinstated significant difference of RR between conventional and Damon self-ligating systems with occurrence ranging between grade 0 and 3 [15]. About 4.1% of patients had an average resorption of at least 1.5 mm of the 4 maxillary incisors, and about 15.5% had 1 maxillary incisor or more with resorption of at least 2.0 mm from 3 to 9 months after initiation of fixed appliance therapy in their study. Although teeth with long, narrow, and deviated roots are at increased risk of resorption during their early stages, the explained variances of these risk factors are less than 25% [16]. Similarly, in this case, mild changes were evident by the end of 13 months that progressed to severe condition by 24 months. In spite of knowing that she is highly susceptible to OIEARR, the best possibility to avoid it was to swiftly reject the treatment with adequate patient education and counseling. Her strong desire to correct the overjet and overbite challenged all the possibilities to proceed with treatment plan.

The pretreatment and posttreatment (Figure 3) OPGs had significant amount of crestal bone loss (CBL) further compromising the bone to root ratio, interpreting collateral bone loss as periodontal fibers are more in the crestal area than the apical region. Birnie estimated that 3 mm of apical root resorption is equal to 1 mm of crestal bone loss thereby adding limitations for restorative therapies also [17]. In this patient with root resorption and crestal bone loss, the maintenance of a generalized healthy periodontal condition is crucial to aid in the longevity of both teeth and improvement in periodontal condition as both were unidirectional and irreparable. The use of probiotics [18] and natural compounds [19] can modify clinical and microbiological parameters in periodontal patients; they could have an effect also in the long-term follow-up of patients presenting RR and CBL. These emerging features could be considered in future clinical trials.

Gingival crevicular fluid markers and salivary markers are also the evolving areas in this field to act much earlier than the radiographic findings [6, 20]. Considering other factors in avoiding root resorption like being selective in bracket prescription, type of brackets, simple wire mechanics, and maintaining light continuous forces along with unavailability of diagnostic technology and poor compliance from doctor-patient aspects made root resorption inevitable in this patient. Over time, many adjunctive therapies with devices were innovated to overcome such collateral encounters like acceleDent [21], microosteoperforation [22] (Propel), low-level laser therapy [23] (LLLT), and low-intensity pulsed ultrasound [24] (LIPUS) to minimize the intensity of root resorption and hasten tooth movement by improving bone remodeling. However, root resorption even with these aids proved not promising with variable results in the literature review [25, 26]. In one of the studies by El-Bialy et al. [27], they revealed the healing effects of LIPUS induced by OIEARR through ultrasound and suggested more intervention studies in this area. Gay et al. [28] in their study on invisalign aligner therapy, being advanced in the field of orthodontic therapy, reported root resorption with an average of less than 10 percent of their original root length in almost every individual.

The ongoing treatment was terminated, and recovery measures were briefed to uplift the self-esteem of the patient. The patient also showed her disinterest to continue the treatment due to personal reasons. Furthermore, to put forward the prognosis was compromised to be very poor with unpredictability of any other future treatment modalities. The objectives of establishing good intercuspal occlusion with dental midline correction and finishing stage high intercuspal contact correction were compromised. Collateral resorption of crestal bone and apical root should be considered while planning prosthetic occlusal rehabilitation with respect to abutment viability and durability.

## 4. Conclusion

Despite considering all incidences and taking preventive measures to avoid those factors that are commonly associated with OIEARR, root resorption was still an inevitable occurrence in this case, which reinstates the fact that the etiology may be beyond mechanical factors and variable tooth morphology. More emphasis should be focused on overall factors that lead to the prevalence of OIEARR and its diagnostic skills. Poor knowledge and understanding of the incidence of root resorption overshadows the narrow perspective of mechanotherapy as a major cause than being one of the causes for root resorption. Indeed, delivering the mechanical forces at optimal thresholds to induce tooth movement to rule out such incidences also remained questionable as it was imminent in this case apart from invariably understanding the role of other factors in its occurrence.

## Figures and Tables

**Table 1:** Archwires used in different stages with time intervals.

S no.	Archwire	Months	Stage
1	0.014^″^ thermal NiTi	4 months	Stage I (15 months)
2	0.016^″^ thermal NiTi	6 months	
3	0.014 × 0.025^″^ thermal NiTi	3 months	
4	0.017 × 0.025^″^ thermal NiTi	2 months	
5	0.019 × 0.025^″^ stainless steel	11 months	Stage II (11 months)

## Data Availability

The photos, x-rays, tracing data used to support the findings of this study are included within the article.
